# Supplementary material for: Rat deconvolution as knowledge miner for immune cell trafficking from toxicogenomics databases
Source: Toxicol Sci. 2023 Nov 6;197(2):121–31. doi: 10.1093/toxsci/kfad117 (PMC10823770; doi:10.1093/toxsci/kfad117)
Supplement: kfad117_Supplementary_Data [file kfad117_supplementary_data.zip › kfad117_Supplementary_Data/toxsci-23-0222-File002.doc]

**Supplementary Figures**

**Supplementary Figure S1**

Comparison of estimated and measured immune cell trafficking in the rat spleen and whole blood cells (WBCs). Values were measured by flow cytometry after cell isolation and red blood cell lysis. Estimated values were calculated by deconvolution using (A) a human-derived signature or (B) mouse-derived signature matrices. Note that these values were converted to *z*-score between the compared samples.

**Supplementary Figure S2**

Comparison of estimated and measured immune cell trafficking in rat liver after compounds were administered. values were measured by FACS after cell isolation and red blood cell lysis. Estimated values were calculated by deconvolution using (A) a human-derived signature matrix or (B) mouse-derived signature matrices. Note that these values were converted to *z*-score between the compared samples. Ctrl, control; APAP, acetaminophen; MDA, methylene dianiline.

**Supplementary Figure S3**

Comparison of estimated and measured immune cell trafficking in rat liver after compounds were administered. Values were measured by FACS after cell isolation and red blood cell lysis. Estimated values were calculated by deconvolution using a rat-specific signature matrix. Note that these values were converted to *z*-score between the compared samples. Ctrl, control; APAP, acetaminophen; MDA, methylene dianiline.

**Supplementary Figure S4**

Comparison of estimated and measured immune cell trafficking in the spleen, the blood or the liver after each compound were administered. Measurement values were measured by the flow cytometry after cell isolation and red blood cell lysis. Estimated values were calculated by deconvolution using a rat-specific signature matrix. Concordance was measured by Pearson correlation (R) and linear regression (dashed line). Note that these values were converted to z-score between the compared samples. Ctrl, control; APAP, acetaminophen; MDA, methylene dianiline.

**Supplementary Figure S5**

(A) Time course of changes in neutrophils estimated in the liver and ALT measured in the blood of rat administered tiopronin as determined from the Open TG-GATEs database. Value of (B) ALT in blood and (C) neutrophils in the liver at 3 h after tiopronin administration. Note that neutrophils values are converted to *z*-score for the corresponding control samples. The tests of significance were conducted using an unpaired sample greater-sided Welch *t* test.

**Supplementary Figure S6**

Comparison of estimated and measured immune cell trafficking in the rat liver after administering each compound. Values were calculated by deconvolution using (A) human-derived signature matrix or (B) mouse-derived signature matrices. Note that these values were converted to *z*-score between the compared samples. Note that each point is the median value in the experiment or in the Open-TGGATEs database (n = 3~4).

**Supplementary Figure S7**

ALT distribution after (A) administration of control solvent or (B) of compound from the Open TG-GATEs database. The vertical dotted line is the threshold for targeting the compounds used for stratification.

**Supplementary Figure S8**

Visualization of immune cell trafficking features of Open TG-GATEs compounds. All features were dimensionally reduced by each method and visualized. These reduced features were subjected to meta-visualization for Figure 4A. Each color corresponds to the color of the stratified compound. LLE, locally linear embedding; MDS, multidimensional scaling; SPCE, spectral embedding; PCA, principal component analysis; TSNE, *t* distributed stochastic neighbor embedding; UMAP, uniform manifold approximation and projection.

**Supplementary Figure S9**

Visualization of transcriptome features of Open TG-GATEs compounds. All features were dimensionally reduced by each method and visualized. These reduced features were subjected to meta-visualization for Figure 4C. Each color corresponds to the color of the compounds stratified by transcriptome analysis. LLE, locally linear embedding; MDS, multidimensional scaling; SPCE, spectral embedding; PCA, principal component analysis; TSNE, *t* distributed stochastic neighbor embedding; UMAP, uniform manifold approximation and projection.

**Supplementary Figure S10**

Time course of changes in immune cell trafficking and ALT in stratified compound clusters. The line is the average value for each cluster, and the filled color around it indicates the 95% confidence interval. Each color corresponds to the color of the compound stratified by analysis of the transcriptome. Note that the scale of the x-axis does not correspond to the actual times.

**Supplementary Figure S11**

Time course of changes in blood biochemistry. The line is the average value for each cluster, and the filled color around it indicates the 95% confidence interval. Each color corresponds to the color of the compound stratified by analysis of immune cell trafficking. Note that the scale of the x-axis does not correspond to the actual times. TBIL, total bilirubin; Plat, Platelets; RBC, red blood cells; LymB, lymphocytes in the blood.

**Supplementary Figure S12**

Time course of changes in single sample gene set enrichment analysis (ssGSEA) scores of the liver transcriptomes. For each cluster, calculated from the immune cell trafficking, from the top to the bottom, the gene ontology terms with the lowest p-value in the comparison test with the other clusters are shown. Terms with (A) highest and (B) lowest ssGSEA score in that time compared with other clusters.

**Supplementary Figure S13**

Time course of changes in single sample gene set enrichment analysis (ssGSEA) scores of the liver transcriptomes. For each cluster, calculated from the liver transcriptomes, from the top to the bottom, the gene ontology terms with the lowest p-value in the comparison test with the other clusters are shown. Terms with (A) highest and (B) lowest ssGSEA score in that time compared with other clusters.

**Supplementary Figure S14**

Time course of changes in single sample gene set enrichment analysis (ssGSEA) scores of the liver transcriptomes. For gene ontology terms of (A) interleukin-1-mediated signaling pathway and (B) platelet aggregation.

**Supplementary Figure S15**

Time course of changes in estimated neutrophils, monocytes, and lymphocytes in the liver using deconvolution and that of measured in the blood of rats administered (A) naphthyl isothiocyanate or (B) thioacetamide as determined from the Open TG-GATEs database. Note that all values were converted to *z*-score for the corresponding control samples. Estimated values for lymphocytes are estimated from sum of NK, B, CD4 T, and CD8 T cells.
